# Sociodemographic and lifestyle correlates of exclusive breastfeeding practices among mothers on antiretroviral therapy in the Eastern Cape, South Africa

**DOI:** 10.1186/s13006-021-00366-4

**Published:** 2021-02-16

**Authors:** Daniel Ter Goon, Anthony Idowu Ajayi, Oladele Vincent Adeniyi

**Affiliations:** 1 Department of Public Health, University of Fort Hare, 5 Oxford Street, East London, 5201 South Africa; 2 Population Dynamics and Sexual and Reproductive Health, African Population and Health Research Centre, APHRC Campus, Manga Close, Nairobi, Kenya; 3 Sociology Department, University of Fort Hare, East London, 5201 South Africa; 4 Department of Family Medicine, East London Hospital Complex, Cecilia Makiwane Hospital, East London, South Africa

**Keywords:** Infants, Exclusive breastfeeding, HIV, Antiretroviral therapy, South Africa

## Abstract

**Background:**

Exclusive breastfeeding (EBF) is associated with a reduction of postnatal HIV transmission and optimal infant growth. Given that the factors influencing exclusive breastfeeding are multi-factorial and context-specific, we examined the prevalence and factors associated with exclusive breastfeeding practice in the first 6 months among mothers on antiretroviral therapy in the Eastern Cape, South Africa.

**Methods:**

This was a cross-sectional study conducted between January to May 2018, on 469 parturient women enlisted in the prevention of mother-to-child HIV transmission cohort study in the Eastern Cape. Mothers were asked to recall whether they breastfed their infant exclusively with breast milk from birth and if so, to state how long they did. We collected relevant sociodemographic, lifestyle, and maternal information by interview. Bivariate and multivariable logistic regression analyses were fitted to determine the sociodemographic and lifestyle factors associated with exclusive breastfeeding practice.

**Results:**

The prevalence of six-month exclusive breastfeeding, measured since birth, was 32.0%. E Exclusive breastfeeding’s prevalence was significantly higher among married women (36.8%), unemployed women (36.6%), non-smokers (32.7%), and those who never drank alcohol (37.0%). Unemployed women (adjusted odds ratio [AOR] 1.66, 95% Confidence Interval [CI] 1.08–2.56) and those with grade 12 or less level of education (AOR 2.76, 95% CI 1.02–7.49) had a higher likelihood of practising EBF for 6 months since birth while mothers who consumed alcohol (AOR 0.54, 95% CI 0.34–0.85) were less likely to practice EBF for 6 months.

**Conclusions:**

The prevalence of six-month exclusive breastfeeding in the study, although comparable with sub-Saharan Africa and worldwide prevalence, remains suboptimal. Advocacy campaigns on EBF must target alcohol cessation and the creation of a favourable workplace environment for lactating mothers.

## Background

Exclusive breastfeeding (EBF) could save the lives of infants and prevent mother-to-child transmission (PMTCT) of HIV infection [1, 2], especially in a country like South Africa with a high HIV burden [3, 4]. Between 2010 and 2015, South Africa has made policy changes in infant feeding practices and PMTCT guidelines. The policy changes resulted in the replacement of free infant formula for EBF for 6 months policy. Mothers are counselled to practice EBF in the first 6 months, continue to breastfeed beyond 6 months whilst introducing complementary feeding. Also, the new policy recommended the adoption of WHO Option B+ (lifelong antiretroviral therapy strategy) for all HIV-infected pregnant and postpartum women, irrespective of their immune suppression status or infant feeding choice [1, 5, 6].

Established global evidence has shown that exclusive breastfeeding prevents both the incidence of and mortality from diarrhoea, which is commonly associated with mixed feeding practices and infant formula feeding practices [7]. Overall, EBF confers the benefits of essential nutrients and robust immune systems, reduction in the risk of malnutrition and diarrheal associated-illnesses, which are the leading causes of infant mortality in developing nations [1, 8–10]. Worryingly, even with available data demonstrating unequivocal benefits of EBF, mothers often do not abide by the recommended 6 months of EBF [11–13], thus, putting infants at high risk of death. The low EBF prevalence is underpinned by several factors, thus suggesting that there is more to learn and understand regarding the prevailing reasons for the reluctance of mothers to breastfeed their infants exclusively. Previous studies conducted in South Africa have indicated contradictory infant feeding messages from older relatives, such as grandmothers, and healthcare workers, low socio-economic indicators, and the fear of transmitting HIV to the infant as among the reasons for mixed-feeding practices [10, 14].

Exclusive breastfeeding prevalences are reportedly low in many countries, despite the manifold benefits accruable from breastfeeding [15–17]. As reported by Victoria et al. [16] study, only 37% of children are exclusively breastfed in developing countries. In sub-Saharan Africa, the prevalence of EBF is 36% [17], which is very low compared with the global EBF target prevalence set at 50% by 2025 [18]. In South Africa, the government effort has yielded some modest improvement in the prevalence of EBF among mothers living with HIV over the years [19]. However, with an estimation of 31.6% EBF prevalence, the country remains below global targets [15, 20, 21]. The fear of HIV transmission and status disclosure is among the reasons for the low prevalence of exclusive breastfeeding [22, 23].

Considering the high HIV epidemic in South Africa, with approximately 7.7 million people with HIV [24], understanding the factors associated with EBF is essential in crafting strategies to support and sustain appropriate breastfeeding practices to increase the prevalence of exclusive breastfeeding. Even though previous studies have examined the prevalence of EBF among lactating mothers living with HIV in South Africa [10, 15, 17, 20, 25–28], scanty information exists in the Eastern Cape, a province with a twin burden of HIV epidemic and poverty. Given that factors affecting EBF practices vary among countries and even within the sub-groups of populations [29], contextual information on the prevalence and determinants of EBF is vital to inform context-specific interventions. Hence, this study seeks to examine the prevalence and the factors influencing EBF practices among mothers receiving antiretroviral therapy (ART) in the Eastern Cape Province, South Africa.

## Methods

### Study design, settings, population, and sample

This cross-sectional study was conducted between January to May 2018 and forms part of a larger project to assess maternal and infant outcomes for the prevention of mother-to-child transmission programme in the Eastern Cape, South Africa. Aspects of the methodology have been previously published elsewhere [30, 31]. Briefly, the target population was parturient women on ART and their infants attending maternity health facilities at Frere, Cecilia Makiwane, and Bisho hospitals. The baseline study was carried out between September 2015 and May 2016 in the Eastern Cape, South Africa. At the time of the data collection, the infant ages range from 18 to 29 months.

All the parturient women on ART (*n* = 1709) gave consent to be contacted for the follow-up study and also provided three phone contacts through which they could be reached. We were successful in reaching 509 contacts. Nine women had died, and six declined to participate in the study, while 26 dropped out of the survey without answering questions on exclusive breastfeeding. Overall, we included 469 participants in this study. The sample of 469 participants is enough to achieve 88% statistical power to detect a difference of 15% in exclusive breastfeeding practice of users and non-users of alcohol, at an alpha value of 0.005 assuming an EBF prevalence of 36% (17).

The Research Ethics Committee of the Walter Sisulu University approved the research protocol. The nature and scope of the study were explained to the participants, whom all gave informed consent before data collection.

### Data collection procedure

Participants were contacted telephonically to participate in this study, using the three phone contacts provided during the baseline survey. Each participant was provided with an option to either visit the nearest hospital for a face-to-face interview or be interviewed via telephone. Two research assistants were trained to complete the interviewer-guided interview using a pre-designed and validated questionnaire. They were trained mainly on the data collection instruments and the sensitivity of the topic under study.

Participants for telephonic interviews were asked to provide a convenient time for the interview. Research assistants could fluently speak and write English and the local language of *IsiXhosa*. A structured questionnaire was designed to obtain information on sociodemographic variables (age, employment, marital status, and level of education), postpartum adherence to ART, and behavioural lifestyles (smoking and alcohol intake).

### Outcome variable

The primary outcome of interest was EBF practice in the first 6 months. Exclusive breastfeeding is defined by WHO as “nothing but breast milk” [2]. This was measured by asking mothers if they initiated EBF for their baby and, if yes, to state the duration of EBF in months. Mothers who breastfed their infants exclusively for 1 to 3 weeks were coded as 1 month.

### Independent variables

The independent variables included in this study were sociodemographic factors, such as age, employment, marital status, level of education, postpartum adherence to ART, and behavioural lifestyle factors such as smoking and alcohol intake. Our variable selection was based on our review of the literature. Age was measured by asking respondents to state their age at their last birthday. Employment was measured by asking if respondents had worked for wages or salary in the past 12 months or not. Also, we asked participants to indicate if they were single, married, cohabiting or divorced. We also asked respondents to indicate the highest level of education they attained.

We defined postpartum adherence to ART as any episode of missed doses of ART since the birth of the index baby. Mothers who had missed taking their ART were classified as non-adherent, and those who reported no missed episode of ART were classified as adherent. Also, we asked respondents to indicate if they had ever used alcohol and if they smoke tobacco products. Responses were classified as “yes” or “no”.

### Statistical analysis

All statistical analyses were carried out using the Statistical Package for Social Sciences (SPSS) for Windows version 24.0 (SPSS, Chicago, IL, USA). We estimated mean, standard deviation, and proportions for all continuous and categorical variables. Inferential statistics (crude and adjusted logistic regression models) were used to examine factors influencing EBF practices among mothers on antiretroviral therapy. We first estimated the unadjusted odds ratio for all sociodemographic, behavioural, and clinical factors. After, we computed an adjusted odds ratio to identify the main factors significantly associated with the practice of exclusive breastfeeding.

## Results

The participants’ mean age was 33 ± 5.7 years. The majority of the participants were 30 years and above (69.3%), single (66.3%), had grade 12 level of education (87.4%), unemployed (61.8%), and were on government social grant (94.5%). Only a few participants smoked (8.1%) or drank alcohol (35.4%) (Table 1).

**Table 1:** Sociodemographic characteristics of participants

Variables	Frequency	Percentage
Age		
24 years and less	33	7.0
25–29 years	111	23.7
30–34 years	137	29.2
35–39 years	121	25.8
40 years and above	67	14.3
Marital status		
Single	311	66.3
Married	125	26.7
Cohabiting	25	5.3
Previously married	8	1.7
Education level		
Grade 7 and less	27	5.8
Grade 8–12	410	87.4
Higher education	32	6.8
Employed in a salary paying job		
Yes	151	32.2
No	318	67.8
Occupation in last 12 months		
Government employee	15	3.2
Non-government employee	112	23.9
Self employed	29	6.2
Student	23	4.9
Unemployed	290	61.8
Receives government social grant		
Yes	443	94.5
No	26	5.5
Smoking		
Yes	38	8.1
No	431	91.9
Drank alcohol in the last year		
Yes	166	35.4
No	303	64.6
Age of index babies at the time of the interview		
18–24 months	118	25.2
25–29 months	281	74.8

### Prevalence of exclusive breastfeeding

The prevalence of six-month EBF was 32.0%. The prevalence of EBF was significantly higher among married women (36.8%; *p* < 0.001), unemployed women (36.6%; *p* < 0.026), non-smokers (32.7%), and those who never drank alcohol (37.0%; *p* < 0.001) (Table 2).

**Table 2:** Correlates of six-month exclusive breastfeeding

Variables	Breastfed exclusively for 6 months ***n*** (%)	Did not breastfeed exclusively for 6 months ***n*** (%)	***P*** - value
Age			
24 years and less	11 (33.3)	22 (66.7)	0.864
25–29 years	38 (34.2)	73 (65.8)	
30–34 years	41 (29.9)	96 (70.1)	
35–39 years	36 (29.8)	85 (70.2)	
40 years and above	24 (35.8)	43 (64.2)	
Marital status			
Single	92 (29.6)	219 (70.4)	0.001
Married	46 (36.8)	79 (63.2)	
Cohabiting	9 (36.8)	16 (64.0)	
Previously married	3 (37.5)	5 (37.5)	
^a^Education level			
Grade 7 and less	9 (33.3)	18 (66.7)	0.170
Grade 8–12	136 (33.3)	237 (66.7)	
Higher education	5 (16.7)	25 (83.3)	
Employed in a salary paying job			
Yes	45 (29.8)	106 (70.2)	0.278
No	105 (33.0)	213 (67.0)	
Occupation in the last 12 months			
Government employee	2 (13.3)	13 (86.7)	0.026
Non-government employee	33 (29.5)	79 (70.5)	
Self employed	4 (13.8)	25 (86.2)	
Student	5 (21.7)	18 (78.3)	
Unemployed	106 (36.6)	184 (63.4)	
Smoking			
Yes	9 (23.7)	29 (76.3)	0.168
No	141 (32.7)	290 (67.3)	
Drank alcohol in the last year			
Yes	38 (22.9)	128 (77.1)	0.001
No	112 (37.0)	191 (63.0)	

^a^Some participants did not provide responses on feeding practices (*n* = 39)

### Factors associated with six-month exclusive breastfeeding practices (multivariate logistic regression analysis)

We selected variables into our model based on our review of the literature of factors shown to influence exclusive breastfeeding. Variables reported to be significant in previous studies were included in our model irrespective of their *p* - values in our bivariate analysis. For example, we adjusted for age even though it did not reach a significant level in our bivariate analysis. We did not include “receiving of social grant” and “engaged in a salary paying job” due to their collinearity with “occupation”. The results of the unadjusted logistic regression analysis indicate that being unemployed (Crude Odds Ratio [COR] 1.77, 95% CI 1.17, 2.68) and having attained grade 12 or less education (COR 2.79, 95% CI 1.06, 7.38) were significantly associated with a higher odds of practising EBF for the first 6 months by the participants (Table 3).

**Table 3:** Crude and adjusted binary logistic regression analysis showing factors associated with 6 months of exclusive breastfeeding

Variables	COR (95% CI)	AOR (95% CI)
Age		
24 years and less	0.90 (0.37, 2.16)	1.12 (0.43, 2.92)
25–29 years	0.93 (0.49, 1.76)	1.02 (0.51, 2.01)
30–34 years	0.77 (0.41, 1.42)	0.73 (0.39, 1.40)
35–39 years	0.76 (0.40, 1.43)	0.68 (0.36, 1.32)
40 years and above	1	1
Marital status		
Single	0.72 (0.48, 1.09)	0.74 (0.48, 1.14)
Married, cohabiting/divorce	1	1
Education level		
Grade 12 and less	2.79 (1.06, 7.38)*	2.76 (1.02, 7.49)*
Higher education	1	1
Occupation in the past 12 months		
Unemployed	1.77 (1.17, 2.68)*	1.66 (1.08, 2.56)*
Employed	1	1
Smoking		
Yes	0.64 (0.29, 1.39)	0.89 (0.39, 2.03)
No	1	1
Drank alcohol in the past year		
Yes	0.51 (0.33, 0.78)*	0.54 (0.34, 0.85)*
No	1	1
Adherence to ART		
No	0.75 (0.49, 1.13)	0.80 (0.52, 1.25)
Yes	1	
Disclosure of status to partner		
No	0.68 (0.39, 1.16)	0.80 (0.45, 1.40)
Yes	1	
Duration of disease (Years)		
5 years and less	1.02 (0.69, 1.50)	1.03 (0.66, 1.62)
Above 5	1	1

*p < 0.05, *COR* Crude Odds Ratio, *AOR* Adjusted Odds Ratio, *CI* Confidence Interval

However, alcohol use was associated with lower odds of practising EBF among the study participants (COR 0.51, 95% CI 0.33, 0.78). The clinical parameters: duration of disease, disclosure of serostatus, and adherence to the current ART had no significant association with EBF practice among the mothers. Similarly, in the adjusted model, being unemployed (AOR 1.66, 95% CI 1.08, 2.56; *p* < 0.05) and having grade 12 or less level of education (AOR 2.76, 95% CI, 1.02, 7.49; *p* < 0.05) were significantly associated with a higher likelihood of exclusive breastfeeding, while alcohol use was significantly associated with lower odds of six-month exclusive breastfeeding (AOR 0.54, 95% CI 0.34, 0.85; *p* < 0.05). The magnitude and the direction of effect persist after adjusting for important demographic, clinical, and behavioural covariates (Table 3).

## Discussion

Our study aimed to examine the prevalence and factors influencing six-month EBF practice among mothers on ART in the Eastern Cape Province, South Africa. Although programmes on 6 months of infant breastfeeding have been developed and approved in South Africa, the uptake of EBF remains low. In this study, the prevalence of six-month EBF of 32.0% was sub-optimal. However, direct comparison of EBF prevalence in this study with many other studies on EBF practice is a difficult task, given the variations in the definitions of EBF in different studies, the timing, duration of recall, methods of analysis, and sample biases [32]. Notwithstanding, the EBF prevalence obtained in this study is comparable to 31.6% among infants exclusively breastfeed for the first 6 months after delivery in Malawi, which was measured by recall method [33].

Generally, the low prevalence of EBF in South Africa is worrying and suggests concerted interventions to promote, support, and protect breastfeeding, beyond policy and programme formulations are needed. Some studies in other provinces in South Africa reported higher EBF prevalence at 43.2% among HIV-positive mothers in Merafong sub-district in Gauteng [34], and 35.6% in Gert Sibande district in Mpumulanga [35]. An earlier study by Siziba et al. [25] reported a low summative prevalence of 12.0% in North-West, Gauteng, Free State, and Eastern Cape Provinces. Also, 18.0, 6.0, 13.0, 7.6, and 6.7% of mothers practiced EBF up to 6 months in Kwa-Zulu Natal, Western Cape, Limpopo and Gauteng provinces, respectively [36–40]. Compared with other settings elsewhere, the EBF prevalence in our study is almost at par with prevalences reported in Ethiopia (30.6, 31.0%) [41, 42], Bangladesh (35.0%) [43] and India (34.0%) [44], but higher than the EBF prevalence reported in Kaiyuan Yunnan, Southwest China (27.34%) [45], Saudi Arabia (24.4%) [46], USA (16.8%) [47], Egypt (9.7%) [48] and Nigeria (14.8%) [49]. Yet, other studies have reported higher EBF prevalence in Ethiopia (88.8, 75.2%; 77.3%) [28, 50, 51], Kenya (71.4%; 52.3%) [52, 53], Western India (61.5%) [54] and Tanzania (55.5%) [55]. The differences in EBF prevalences reported across various countries or regions in the literature could be explained in the light of differences in the definition of EBF and geographic variations in the interplay of cultural, economic, and sociodemographic factors affecting exclusive breastfeeding.

Notwithstanding the many advantages of breastfeeding and the strategies to promote it, EBF uptake still remains low in many developing countries [16, 56]. Worryingly, worldwide, only 35% of infants are exclusively breastfed [57]. Exclusive breastfeeding is associated with a reduction in child mortality in low-income countries [58, 59]; thus, interventions on EBF should be accorded top priority. In this regard, there is a need to sensitise the community on the benefits and inherent problems associated with mixed feeding [47]. In the South African context, one of the pragmatic approach to promote women’s awareness of EBF, outside of health facility channels, could be to utilise and encourage the Ward-Based-Outreach-Teams (WBOT) in the community or women’s groups to increase the duration of EBF. One of the cardinal objectives of WBOT is to promote and create awareness on various health issues affecting the community as part of the government efforts of improving the primary healthcare re-engineering agenda. This is advisable because community beliefs could have considerable influence on EBF practice.

Our findings demonstrated that being unemployed and having a low level of education (secondary or less) was significantly associated with a higher likelihood of exclusive breastfeeding. In contrast, alcohol use was significantly associated with lower odds of six-month exclusive breastfeeding practice. Previous studies have linked maternal employment with lower prevalences of EBF and earlier cessation of breastfeeding [60, 61]. In this study, consistent with studies conducted in Bangladesh [43], Saudi Arabia [46], Ethiopia [62–64], Tanzania [65], Canada [66], and Guatemala [67], unemployed mothers are likely to practise EBF as compared to those who are employed. It is plausible that mothers who are not employed do have enough opportunity while at home to breastfeed their infants. Contrastingly, employed mothers, perhaps due to the nature of their work with the challenge to return early to work after giving birth, work shifts, and maternal fatigue, may collectively hinder them from having frequent contact with their infant to provide exclusive breastfeeding. In South Africa, working mothers are granted only 4 months of maternity leave, which may begin at any time from at least 4 weeks before the birth of the baby, and there are no available workplace facilities for mothers to breastfeed children. The Code of Good Practice on the protection of employees during pregnancy and after childbirth included in the Basic Conditions of Employment Act (Republic of South Africa, 1997), stipulated that arrangement should be made to accommodate employees who are breastfeeding, with 30 min breastfeeding breaks twice a day to breastfeed or express for the first 6 months of the child’s life (paragraph 5.13) (the Republic of South Africa, Department of Labour, 1998). However, the actual situation is that women employees and their employers are mostly unaware of this legislation on breastfeeding breaks [68, 69], which are seldom provided or requested in the workplace. Policies about maternity leave in South Africa warrant scrutiny to encourage EBF practice. This is very crucial in the context of achieving comprehensive PMTCT.

In this study, mothers with a low level of education are inclined to practise exclusive breastfeeding. Previous studies have reported similar findings [43, 70–73]. However, Mango et al. [16] study in Tanzania found the level of education had no association with EBF practice. Other studies conducted in Ethiopia and Bangladesh reported similar findings [74, 75]. The association of a lower level of education with EBF could be explained by the higher rate of unemployment in this population. As such, strategies aimed at strengthening EBF practise in this population should also address the needs of women with higher levels of education. Specifically, the concerns and fears of educated women need to be addressed during counselling sessions at antenatal and postnatal clinics.

This study revealed that alcohol use was significantly associated with lower odds of six-month exclusive breastfeeding practice. About 22.9% of mothers drink alcohol. A survey conducted in the Kilimanjaro region, Tanzania, showed that mothers’ alcohol intake was associated with EBF up to 6 months [73]. Other studies have reported similar findings elsewhere [70–72, 76]. However, Mgongo et al. [56] reported a contrary result. Previous studies have linked alcohol intake with HIV and poor child immunisation [77, 78]. A recent study has reported high binge drinking (10.8%) among women in South Africa [79]. Alcohol consumption is widely practised in South Africa [79], at various events or celebrations such as weddings, burials, and other social events, work or community engagements. Advocacy programmes on the effects of alcohol intake during lactating are crucial. Alcohol use, whilst breastfeeding, has negative effects on newborns, EBF, and general infant growth [80, 81].

### Limitations

Though the measure of EBF (nothing but breast milk) and its duration was clearly explained to the mothers, we could not ascertain if there was any confusion between maternal definitions of EBF and the EBF definition in this study. Often, retrospective data collection tends to result in overestimation of the prevalence and duration of EBF practice in general [32]. Hence, the extent of recall bias and social desirability bias cannot be ascertained, given that this study was a follow up of an existing cohort, which occurred between 18 to 29 months after the delivery of the index infants. Also, telephonic survey with its inherent challenges might have impacted the responses of the parturient women. Regardless of these limitations, our study provides useful information for future comparative studies on the factors influencing infant-feeding practices by mothers on antiretroviral therapy in the Eastern Cape Province, South Africa. Such information would be relevant in shaping maternal and child health interventions in the context of paediatric HIV infection, at least in this setting. A qualitative study would provide better insight and understanding of the reasons why the majority of the women did not practise EBF in the region.

## Conclusions

The prevalence of exclusive breastfeeding in the study, although comparable with sub-Saharan Africa and worldwide prevalence, remains suboptimal. Interventions aimed at promoting, supporting, and protecting breastfeeding beyond policy and programme formulations are urgently needed in the region. The factors affecting the EBF of HIV-positive mothers in this study include employment, low levels of education, and alcohol consumption. A general advocacy campaign on exclusive breastfeeding must target alcohol cessation and the creation of a favourable workplace environment for lactating mothers.

## Data Availability

Data included in this study are available upon reasonable request from the corresponding author.

## Abbreviations

AOR: Adjusted Odds Ratio
ART: Anti-Retroviral Therapy
COR: Crude Odds Ratio
CI: Confidence Interval
EBF: Exclusive Breastfeeding
PMTCT: Prevention of Mother-to-Child Transmission
WBOT: Ward-Based-Outreach-Teams
WHO: World Health Organization
